# The oral-gut microbiota axis in cardiovascular diseases: mechanisms, therapeutic targets, and translational challenges

**DOI:** 10.3389/fcimb.2025.1658502

**Published:** 2025-09-25

**Authors:** Shuling Su, Xiaobin Ni, Yongluan Lin

**Affiliations:** ^1^ Department of Cardiology, The First Affiliated Hospital of Shantou University Medical College, Shantou, Guangdong, China; ^2^ Shantou University Medical College, Shantou, Guangdong, China; ^3^ Key Laboratory for Prevention and Control of Arrhythmia and Panvascular Disease, Shantou, Guangdong, China

**Keywords:** oral-gut microbiota axis, cardiovascular diseases, microbial translocation, metabolic cross-talk, immune activation

## Abstract

The oral-gut microbiota axis, a newly recognized regulatory system, has emerged as a pivotal factor in the development of cardiovascular diseases (CVDs). This review comprehensively synthesizes the latest evidence on how the dysbiosis of oral and gut microbiota, along with their metabolic and immunological cross-talk, contributes to CVD pathogenesis, including atherosclerosis, hypertension, and heart failure. We highlight the novel “microbiota-metabolism-immunity”tri-dimensional regulatory network and explore innovative therapeutic strategies, such as precision microbiome modulation and non-invasive biomarker development. By bridging the gap between basic research and clinical translation, this review provides new insights into preventing and treating CVDs through targeting the oral-gut axis.

## 1 Introduction

Cardiovascular diseases (CVDs) remain the predominant global cause of mortality, contributing to approximately 32% of all fatalities (World Health Organization (WHO)). Traditional risk factors, such as smoking, hypertension, and hyperlipidemia, have been well-established; however, the role of the microbiota in CVD development has gained increasing attention in recent years. The concept of the “oral-gut microbiota axis,” defined by Liu et al. in 2025 (Xu et al., 2025), describes a trans-organ regulatory system interconnected by microbial translocation, metabolic cross-talk, and immune signaling.

Previous studies primarily focused on the association between periodontitis and CVDs, overlooking the complex interplay between oral and gut microbiota. For instance, a large-scale cohort study from the CoLaus|PsyCoLaus project (N = 3459) revealed that antibodies against *Fusobacterium nucleatum*, a common oral pathogen, were significantly associated with an increased risk of cardiovascular events (Hodel et al., 2023). A meta-analysis study containing 31 studies comprising of 11,132 human samples from 2008 to 2022 reported that dysbiosis of *phyla Proteobacteria*, *Firmicutes*, and *Bacteroidetes* in blood circulation which could be colonized in the gastrointestinal tract, was associated with cardio-metabolic diseases (Goraya et al., 2022; Ullah Goraya et al., 2023). Besides, a cross-sectional and a 6-month follow-up study to analyze the roles of oral and gut microbiota in hypertension discovered that Ectopic colonization of saliva-derived *Veillonella* in the gut might aggravate hypertension (Chen et al., 2023). Thess evidences underscored the urgency of exploring the oral-gut axis as a potential therapeutic target for CVDs.

## 2 The oral and gut microbiota: composition and functional crosstalk

### 2.1 Oral microbiota landscape

The oral cavity, composed of the anterior lip, the lateral cheek, the floor of the inferior mouth, the posterior oropharynx, the superior palate and characterized by its unique microenvironment with varying pH levels, oxygen concentrations, and nutrient availability, harbors a highly diverse microbial community (Madani et al., 2014). Metagenomic studies have identified over 700 species, with *Firmicutes* and *Bacteroidetes* constituting the dominant phyla (Schamarek et al., 2023). Among them, *Streptococcus* spp. within *Firmicutes* are often found in the early stages of dental plaque formation, adhering to tooth surfaces through surface proteins and polysaccharides, forming a biofilm that provides a protective niche for other bacteria (Nobbs et al., 2009). By contrast, *Prevotella* spp. from the *Bacteroidetes* phylum exhibit higher prevalence in periodontal pockets and are strongly contributed to the exacerbation of periodontal diseases (Ge et al., 2013).

*Pathogenic* species like *Porphyromonas gingivalis* play a critical role in oral dysbiosis. This anaerobic bacterium secretes virulence factors, such as gingipains, which are cysteine proteases. Gingipains degrade various components of the oral epithelial barrier, including collagen, fibronectin, and laminin, weakening the tissue integrity (Jayaprakash et al., 2014). Additionally, they can cleave host cytokines and chemokines, modulating the local immune response to facilitate bacterial survival and invasion. It was demonstrated in a 2019 study that salivary *Porphyromonas gingivalis* abundance is associated with the onset and severity of periodontitis (Damgaard et al., 2019).

### 2.2 Gut microbiota architecture

The gut microbiota exhibits significant spatial heterogeneity along the gastrointestinal tract. In the ileum, due to faster transit time and higher oxygen levels, microbial density is lower, and the community structure differs markedly from that of the colon. Certain *Firmicutes* species are present in the ileum; however, in the colon, anaerobic *Firmicutes* efficiently ferment complex carbohydrates into short-chain fatty acids (SCFAs) like butyrate. Butyrate serves as a primary energy source for colonic epithelial cells and possesses anti-inflammatory properties. In contrast, the colon, characterized by slower transit and an anaerobic environment, harbors a higher microbial density dominated by *Firmicutes* and *Bacteroidetes*. *Bacteroidetes* species excel at degrading dietary fibers that resist digestion in the upper gastrointestinal tract, producing SCFAs and other metabolites (Zhang et al., 2014; Zhang et al., 2018; Martinez-Guryn et al., 2019).

Studies have shown that the composition of the gut microbiota is closely associated with host metabolism. In metabolic disorders such as obesity, gut microbial diversity is often reduced, and the relative abundance of certain bacterial groups is altered. Early research suggested that the ratio of *Firmicutes* to *Bacteroidetes* may be elevated in obese individuals, potentially reflecting an increased capacity to extract energy from the diet (Ley et al., 2006). However, subsequent studies have questioned the consistency of this ratio as a reliable biomarker for obesity, as it is significantly influenced by factors such as diet, ethnicity, and lifestyle (Sze and Schloss, 2016; Magne et al., 2020). As a result, current research increasingly emphasizes the functional characteristics of the gut microbiota and the associated metabolic pathways, rather than relying solely on specific taxonomic ratios to assess metabolic health.

One of the most well-studied gut microbiota-derived metabolites in the context of cardiovascular diseases is trimethylamine N-oxide (TMAO). Gut microbes can convert dietary phosphatidylcholine, choline, and L-carnitine into trimethylamine (TMA), which is then absorbed into the bloodstream and oxidized in the liver by the enzyme flavin-containing monooxygenase 3 (FMO3) to form TMAO. Multiple prospective cohort studies have shown that elevated TMAO levels are significantly associated with an increased risk of cardiovascular events, including myocardial infarction, stroke, and cardiovascular death (Koeth et al., 2013; Schiattarella et al., 2017). A meta-analysis further revealed that for each 1 μmol/L increase in TMAO concentration, the risk of cardiovascular events rises by approximately 23% (Tang et al., 2013). Mechanistic studies suggest that TMAO promotes the development and progression of atherosclerosis by enhancing macrophage cholesterol uptake, inhibiting cholesterol efflux, and inducing endothelial dysfunction.

### 2.3 Cross-talk mechanisms between oral and gut microbiota

#### 2.3.1 Microbial translocation

Recent research has made significant progress in elucidating the mechanisms by which oral bacteria translocate to the gut. A study published in Gut Microbes in 2024 demonstrated that oral pathobiont *Klebsiella* spp. can specifically adhere to inflamed intestinal mucosa via chaperone-usher pili (CUP), a surface protein structure (Guo et al., 2024). During conditions such as inflammatory bowel disease or after antibiotic treatment, the intestinal epithelial barrier becomes more permeable, allowing oral bacteria swallowed during normal digestion to cross the compromised barrier and colonize the gut lumen. Once established, these oral bacteria can disrupt the gut microbial balance, leading to a reduction in beneficial short-chain fatty acid (SCFA)-producing bacteria and an increase in pro-inflammatory cytokines. This imbalance may promote the translocation of bacterial lipopolysaccharides (LPS) into the bloodstream, triggering systemic inflammatory responses (Apoorva, 2020; Guo et al., 2024).

#### 2.3.2 Metabolic cross-feeding

There may be a metabolic cross-feeding relationship between the oral and gut microbiota. Although direct evidence is currently lacking to confirm a significant positive correlation between salivary lactate produced by oral lactic acid bacteria and fecal short-chain fatty acids (SCFAs) synthesized by gut bacteria, existing studies have shown that lactic acid bacteria can influence the composition and metabolic activity of the gut microbiota (Li et al., 2024). Lactic acid bacteria ferment dietary carbohydrates into lactate, which can be further metabolized by certain gut bacteria—such as *Veillonella* and *Bacteroides*—into acetate and propionate. Propionate has been demonstrated to modulate host energy metabolism and inflammation by activating G protein-coupled receptors (e.g., *GPR41* and *GPR43*) on immune cells, thereby inhibiting the *NF-κB* signaling pathway and reducing the production of pro-inflammatory cytokines (Akhtar et al., 2022). In addition, propionate can stimulate the secretion of glucagon-like peptide-1 (*GLP-1*) from intestinal endocrine cells, contributing to improved glucose homeostasis and potentially protecting cardiovascular health by reducing insulin resistance (Kimura et al., 2011).

Notably, recent research has begun to reveal the unique role of gut fungi in the metabolism-inflammation axis. Cross-kingdom interactions between fungi and bacteria also show potential synergistic pathogenic mechanisms. For instance, when *Candida albicans* and *Streptococcus mutans* coexist in dental plaque biofilms, they enhance biofilm structural stability and pro-inflammatory capacity (Falsetta et al., 2014; Ellepola et al., 2019). This “fungus-bacterium interaction” mechanism has recently been proposed as a potential new driver of inflammatory diseases, including cardiovascular conditions. Therefore, exploring the metabolic cross-feeding and inflammatory synergy between fungi and bacteria within oral and gut ecosystems may provide new insights into the pathogenesis of systemic metabolic diseases.

## 3 The oral-gut axis in cardiovascular pathogenesis

### 3.1 Atherosclerosis: from plaque initiation to instability

The Oral–Gut Microbiota Axis Plays a Pivotal Role in Multiple Stages of Atherosclerosis, Including Plaque Formation, Cholesterol Metabolism Disorders, and Plaque Instability Leading to Thrombosis.

#### 3.1.1 Plaque formation and immune activation

In the early stages of the disease, oral pathogens such as *Porphyromonas gingivalis* and *Fusobacterium nucleatum* may be swallowed through the oropharynx and enter the gut, where they disrupt the balance of the gut microbiota. For instance, colonization by *F. nucleatum* has been associated with a reduction in *Lactobacillus* populations (Xie et al., 2024), accompanied by decreased levels of anti-inflammatory cytokines (e.g., IL-10) (Zhao et al., 2019) and increased levels of pro-inflammatory cytokines (e.g., IL-17, TNF-α) (Chen et al., 2015; Gebremariam et al., 2019). IL-17 can activate vascular endothelial cells and upregulate the expression of *VCAM-1* and *ICAM-1*, thereby promoting immune cell adhesion and migration to the vessel wall, creating a pro-inflammatory environment conducive to plaque formation (Mai et al., 2016). Notably, recent studies have provided direct evidence of oral pathogens in atherosclerotic tissue. In 2017, J-L C Mougeot and colleagues used 16S rRNA gene-based metagenomic analysis to identify 230 species of oral microbiota within atherosclerotic plaques, with *P. gingivalis* being the most abundant (Mougeot et al., 2017). This supports a potential causal relationship between oral infections, atherosclerosis, and cardiovascular events.

#### 3.1.2 Cholesterol metabolism disorders

The oral–gut microbiota axis also plays a crucial role in cholesterol metabolism. Trimethylamine-N-oxide (TMAO), a metabolite produced by gut bacteria from dietary choline and carnitine, has been closely linked to atherosclerosis. Studies have shown that TMAO can inhibit the expression of cholesterol transporters *ABCA1* and *ABCG1* in macrophages, reducing cholesterol efflux and leading to intracellular cholesterol accumulation and foam cell formation—a hallmark of atherosclerotic plaques (Luo et al., 2024). Additionally, TMAO can activate the *TLR4/NF-κB* signaling pathway, thereby promoting inflammatory responses and accelerating atherosclerosis progression (Hakhamaneshi et al., 2021). Interestingly, oral bacteria such as *Prevotella* and *Fusobacterium* are also capable of producing TMA, suggesting that the oral microbiome may indirectly contribute to atherosclerosis by influencing TMAO production (Nakajima et al., 2015).

#### 3.1.3 Plaque instability and thrombosis

In the later stages of atherosclerosis, the oral–gut microbiota axis may contribute to plaque instability and thrombosis through systemic inflammation and metabolic disruption. Chronic periodontitis can lead to oral pathogens such as *P. gingivalis*, or its virulence factors (e.g., lipopolysaccharide [LPS] and gingipains), being swallowed into the gut, where they disrupt the microbial ecology. Studies have shown that such dysbiosis increases pro-inflammatory bacteria like *Enterobacteriaceae* and *Fusobacterium*, while decreasing barrier-protective and butyrate-producing bacteria. This induces increased intestinal permeability (“leaky gut”), allowing pathogen-associated molecular patterns such as LPS to more easily enter the circulation, triggering systemic inflammation (Nakajima et al., 2015; Sun et al., 2024). This systemic inflammation can activate matrix metalloproteinases (MMPs) within atherosclerotic plaques, leading to degradation of collagen and elastin, thereby weakening plaque structure (Di Nubila et al., 2024). In addition, circulating oral-derived toxins (e.g., P. gingivalis LPS) can activate endothelial cells and platelets via the *TLR4/NF-κB* signaling pathway, promoting platelet adhesion, aggregation, and inflammation. In recent years, P. gingivalis DNA has also been detected in coronary thrombi of patients with acute myocardial infarction (Piñon-Esteban et al., 2020; Joshi et al., 2022), and its secreted gingipains can activate protease-activated receptors (*PAR-1* and *PAR-4*) on platelets, inducing aggregation and pro-inflammatory cytokine release (Khalaf et al., 2017). Its lipopolysaccharides may also activate immune cells through the *TLR4/NF-κB* pathway (Khalaf et al., 2017). These dual mechanisms may promote thrombosis and exacerbate atherosclerosis. Therefore, the oral–gut microbiota axis not only contributes to systemic inflammation through local infections but also disrupts gut barrier function and microbiota composition, promoting a pro-inflammatory vascular environment and platelet activation, potentially serving as a critical driver of plaque rupture and acute cardiovascular events.

### 3.2 Hypertension: the role of gut-derived metabolites

The oral-gut microbiota axis is increasingly recognized as a contributing factor in the development of hypertension, primarily through its regulation of gut-derived metabolites and immune responses.

#### 3.2.1 Intestinal barrier dysfunction and endotoxemia

Oral microbiota dysbiosis may lead to alterations in gut microbial composition, subsequently affecting the integrity of the intestinal barrier (Murugesan and Al Khodor, 2023). When this barrier is compromised, lipopolysaccharide (LPS) from the outer membrane of Gram-negative bacteria can enter systemic circulation, resulting in endotoxemia. LPS activates the Toll-like receptor 4 (*TLR4*) signaling pathway, inducing the production of proinflammatory cytokines and reactive oxygen species (ROS) in vascular endothelial and smooth muscle cells. The increase in ROS impairs endothelial nitric oxide synthase (eNOS) function, reducing nitric oxide (NO) production, a key vasodilator. The subsequent vasoconstriction and increase in peripheral vascular resistance ultimately lead to elevated blood pressure (Dinakis et al., 2024).

#### 3.2.2 Activation of the renin-angiotensin system

The oral-gut axis can also activate the renin-angiotensin system. Gut microbiota-derived short-chain fatty acids (SCFAs), such as propionate and butyrate, modulate the RAS via G protein-coupled receptors (e.g., *GPR41, GPR43*, and *Olfr78*). For instance, propionate activates *GPR41* to promote vasodilation and lower blood pressure, whereas *Olfr78* activation may increase renin release and raise blood pressure. SCFAs also regulate renal gene expression of renin and angiotensinogen, thereby affecting RAS activity (Pluznick et al., 2013; Gotoh and Shibata, 2023). Furthermore, systemic inflammation caused by oral and gut dysbiosis can stimulate the release of angiotensin-converting enzyme (ACE) (Nobbs et al., 2009; Ge et al., 2013), leading to RAS activation, sodium and water retention, increased blood volume, and eventually elevated blood pressure (Schenkein and Loos, 2013; Santisteban et al., 2017).

#### 3.2.3 The gut-brain axis and hypertension

Notably, in the past five years, the role of the gut-brain axis in hypertension—particularly resistant hypertension—has gained increasing attention. Metabolites produced by gut microbiota, such as SCFAs, can influence the central nervous system via the vagus nerve, regulating sympathetic nervous activity and thereby affecting blood pressure. In addition, gut dysbiosis may increase proinflammatory cytokines such as IL-17 and TNF-α, which can activate central nervous system inflammation and further promote the development of hypertension (Cui et al., 2024).

### 3.3 Heart failure: bidirectional regulation and controversies

The oral-gut microbiota axis plays a complex and bidirectional role in the pathogenesis of heart failure, exerting both potentially harmful effects and compensatory adaptive responses.

#### 3.3.1 Microbial dysbiosis and the progression of heart failure

A 2024 review published in the International Journal of Molecular Sciences highlights that dysbiosis of the oral microbiota is closely associated with the development of cardiovascular diseases (CVD) (Mai et al., 2016). Oral pathogens and their metabolites can enter the bloodstream either directly through the oral mucosa or indirectly by translocating to the gut, thereby triggering systemic inflammatory responses. In patients with heart failure, such inflammation may further impair cardiac function. For example, the presence of pathogenic bacteria such as *Porphyromonas gingivalis* is associated with elevated levels of high-sensitivity C-reactive protein (hs-CRP) and other pro-inflammatory cytokines, which may contribute to myocardial fibrosis, reduced cardiac contractility, and decreased cardiac output.

#### 3.3.2 Compensatory mechanisms and microbiota adaptation

A 2023 study published in the American Journal of Physiology – Heart and Circulatory Physiology observed compositional shifts in the gut microbiota of heart failure patients, characterized by a reduction in beneficial bacteria and an increase in pathogenic ones (Ahmad et al., 2023). The study also reported a decline in short-chain fatty acids (SCFAs) such as butyrate, which may be associated with disease progression. However, in some cases, these microbiota changes may represent an adaptive response aimed at maintaining host metabolic and immune homeostasis in the context of heart failure.

#### 3.3.3 Strain-specific function hypothesis

Notably, certain bacterial genera exhibit “double-edged sword” effects in heart failure. For instance, *Akkermansia muciniphila* is widely recognized for its metabolic benefits: it strengthens the intestinal mucus layer, modulates immune responses, and produces anti-inflammatory metabolites such as SCFAs, which can improve metabolic profiles and endothelial function (Everard et al., 2013). However, under pathological conditions such as compromised intestinal barriers or inflammation, *A. muciniphila*’s mucin-degrading activity may increase gut permeability, facilitate the translocation of pathogens like *Salmonella*, and trigger systemic inflammation, potentially exacerbating cardiovascular damage (Hikima et al., 2021).

In light of these findings, the “strain-specific function hypothesis” has been proposed: different strains of the same microbial species may exert markedly different, even opposing, physiological effects. For example, *A. muciniphila* strains vary significantly in membrane protein expression, immune modulation capacity, and metabolite production. Some strains may induce protective immune responses, while others could be pathogenic (Zhang and Zhao, 2016). Therefore, future microbiota-based therapies should shift from a species-level to a strain-level precision design, in order to enhance therapeutic efficacy and avoid potential risks.

## 4 “Microbiota-metabolism-immunity” tri-dimensional regulatory network

**Figure 1:**
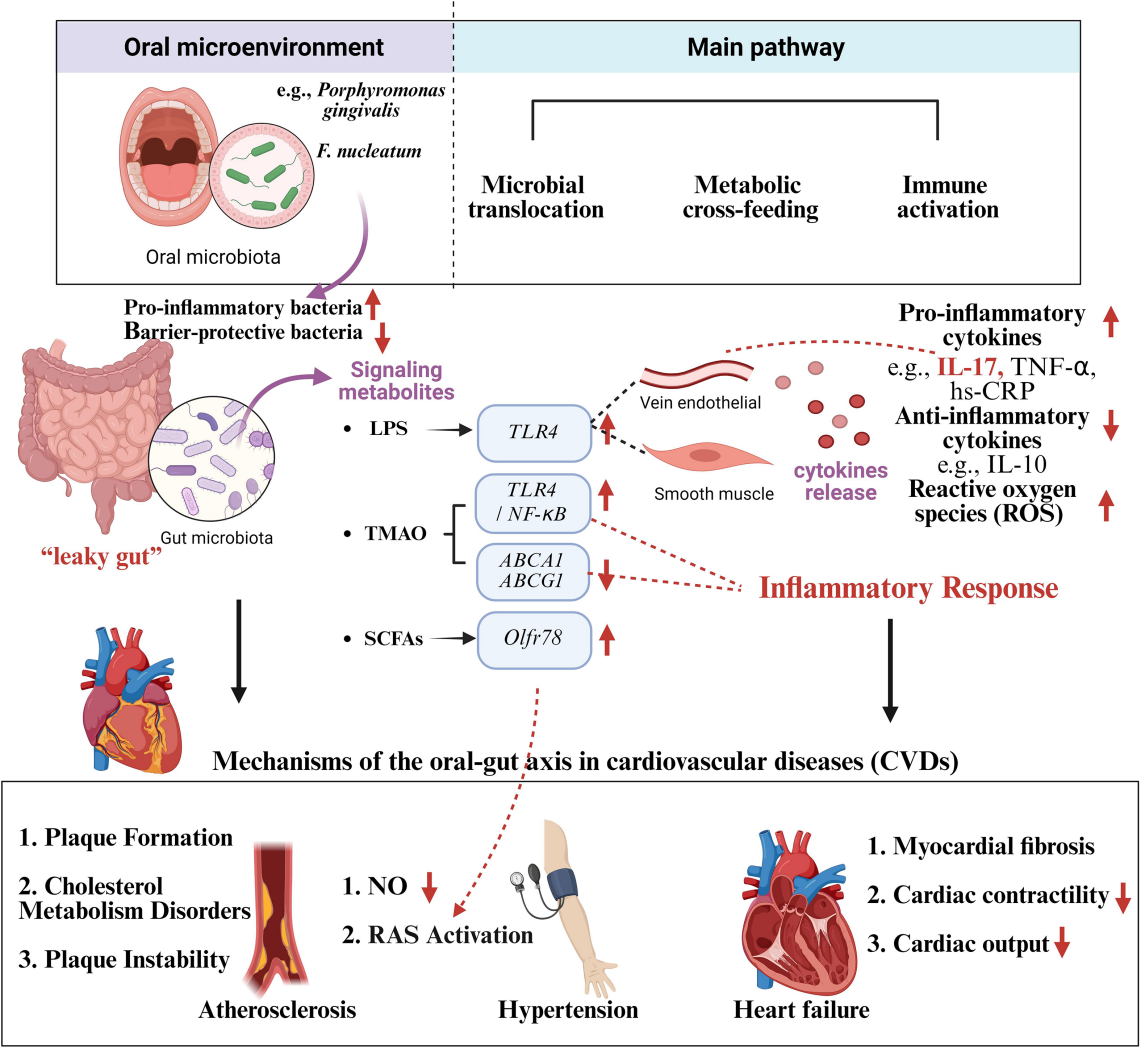
Mechanisms of the oral-gut axis in cardiovascular diseases (CVDs). Oral dysbacteriosis affects the gut microbiota and triggers systemic inflammation through microbial translocation, metabolic cross-feeding and immune activation, leading to cardiovascular diseases such as atherosclerosis, hypertension and heart failure. Created in BioRender. Su, S. (2025) https://BioRender.com/sbc50mx.

According to what have been above, “microbiota-metabolism-immunity” tri-dimensional regulatory network was constructed to describe how the dysbiosis of oral and gut microbiota, along with their metabolic and immunological cross-talk, contributes to CVD pathogenesis. “Microbiota” referring to microbial translocation means that oral bacteria can cross the compromised barrier and colonize the gut lumen. “Metabolism” referring to metabolic exchange means that there may be a metabolic cross-feeding relationship between the oral and gut microbiota. “Immunity” refers to immune activation of pro-inflammatory cytokines and immune signaling pathway to cultivate pro-inflammatory environment. Microbial translocation can disrupt oral-gut microbial homeostasis, triggering systemic inflammation and promoting CVD. Microbial metabolites may mediate immune activation and dysbacteria, leading to CVD. They are interrelated and interact on each other, contributing to CVD together (**Figure 1**, **Table 1**).

**Table 1:** Mechanisms of action of CVD‐associated oral-gut bacterial species.

Organism	Effect in CVD	Mechanism
*Porphyromonas gingivalis*	Atherosclerosis: Plaque Instability and Thrombosis	Increase pro-inflammatory bacteria Decrease barrier-protective and butyrate-producing bacteria Increase intestinal permeability to systemic inflammation and metabolic disruption Activate *TLR4/NF-κB* pathway to promote platelet adhesion, aggregation, and inflammation Activate *PAR-1* and *PAR-4 to i*nduce aggregation and pro-inflammatory cytokine release
	Hypertension: Intestinal Barrier Dysfunction and Endotoxemia	LPS activate *TLR4* pathway: Increase ROS to reduce NO
	Hypertension: Activation of the RAS	Release of ACE to RAS activation
	Heart failure: Myocardial fibrosis, reduced cardiac contractility, and decreased cardiac output	Elevate hs-CRP and pro-inflammatory cytokines
*Fusobacterium nucleatum*	Atherosclerosis: Plaque Formation and Immune Activation	Decrease anti-inflammatory cytokines (e.g., IL-10) Increasd pro-inflammatory cytokines (e.g., IL-17, TNF-α) Upregulate *VCAM-1* and *ICAM-1* Promote immune cell adhesion and migration to the vessel
	Atherosclerosis: Cholesterol Metabolism Disorders Plaque Instability and Thrombosis	Produce TMA: Inhibit *ABCA1* and *ABCG1* Cholesterol accumulation and foam cell formation Activate the *TLR4/NF-κB* pathway Promote inflammatory responses
	Hypertension: Intestinal Barrier Dysfunction and Endotoxemia	LPS activate TLR4 pathway: Increase ROS to reduce NO
	Hypertension: Activation of the RAS	Produce SCFAs: Olfr78 activation to increase renin release Release of ACE to RAS activation
*Prevotella*	Atherosclerosis: Cholesterol Metabolism Disorders	Produce TMA: Inhibit *ABCA1* and *ABCG1* Cholesterol accumulation and foam cell formation Activate the *TLR4/NF-κB* pathway Promote inflammatory responses
	Hypertension: Intestinal Barrier Dysfunction and Endotoxemia	LPS activate TLR4 pathway: Increase ROS to reduce NO
	Hypertension: Activation of the RAS	Produce SCFAs: Olfr78 activation to increase renin release Release of ACE to RAS activation
*Akkermansia muciniphila*	Heart failure: Strain-specific function hypothesis	Strengthen the intestinal mucus layer, modulate immune responses Mucin-degrading activity Increase gut permeability Trigger systemic inflammation

CVD, cardiovascular diseases; TLR4, Toll-like receptor 4; NF-κB, nuclear factor-kappa B; PAR-1, Protease-activated receptor-1; PAR-4, Protease-activated receptor-4; VCAM-1, Vascular cell adhesion molecule 1; ICAM-1, Intercellular adhesion molecule 1; ABCA1, ATP-binding cassette transporter A1; ABCG1, ATP Binding Cassette Subfamily G Member 1; Olfr78, Olfactory receptor 78; ROS, reactive oxygen species; NO, nitric oxide; RAS, renin-angiotensin system; ACE, Angiotensin converting enzyme; hs-CRP, hypersensitive C-reactive protein; IL-10, interleukin-10; IL-17, interleukin-17; TNF-α, tumor necrosis factor-α; TMA, trimethylamine; LPS, Lipopolysaccharide; SCFAs, short-chain fatty acids.

## 5 Oral-gut axis therapeutic strategies

### 5.1 Precision microbiome modulation

#### 5.1.1 Phage therapy

Bacteriophages are viruses that specifically infect and lyse certain bacteria and have been increasingly studied as a targeted antimicrobial therapy alternative to antibiotics. In studies on the oral-gut axis, phages have been designed to eliminate oral pathogens that promote cardiovascular diseases (CVD), such as *Fusobacterium nucleatum* (Kowalski et al., 2022) and *Porphyromonas gingivalis* (Brodala et al., 2005; Matrishin et al., 2023).

Research has found that *P. gingivalis* harbors prophages, suggesting that its ecological system may be regulated by phages (Matrishin et al., 2024). A 2019 study discussed the CRISPR-Cas system in *P. gingivalis*, focusing on how it protects against phage infection through adaptive immune mechanisms within periodontal pockets (Chen and Olsen, 2019). A 2023 study analyzed the CRISPR-Cas systems of “red complex” bacteria associated with periodontitis (including *P. gingivalis*) and found that these systems may help bacteria resist phage infection by identifying and neutralizing phage DNA (Yadalam et al., 2023). Therefore, engineering phages using CRISPR-Cas systems to enhance their specificity and effectiveness against *P. gingivalis* is becoming a cutting-edge direction in precision microbial intervention research.

Since phages can kill bacteria within minutes, phage therapy may lead to endotoxin release rapidly and cause adverse reaction including hypersensitivity and cytokine release syndromes (Liu et al., 2021). An case report in German Heart Center Berlin showing 3 of 4 patients with cardiovascular implant infections were infection free after bacteriophage therapy, indicated phage therapy could be applied as an alternative strategy for patients with chronic relapsing cardiovascular implant infections (Tkhilaishvili et al., 2022). No adverse events related to phage application were reported in the first safety trial in England about bioavailability of oral phage in humans (Bruttin and Brüssow, 2005). Besides, a retrospective observational study including 100 cases of bacteriophage therapy in Belgium reported that more that 70% patients with clinical improvement but there were 15 adverse events including seven non-serious adverse drug reactions suspected to be linked to bacteriophage therapy (Pirnay et al., 2024). Further clinical trials are needed to assess the safety of phage therapy application.

#### 5.1.2 Combined intervention therapy

In recent years, combined intervention strategies have also emerged. In the 2019 review article Phage Therapy: A Renewed Approach to Combat Bacterial Infections published in Cell Host & Microbe, Kortright et al. highlighted the mechanistic differences between phages and traditional antibiotics and proposed that their combination might yield synergistic effects (Kortright et al., 2019). They also suggested that prebiotics and probiotics could enhance the efficacy of phage therapy by modulating the host microbiota. Although this review was largely theoretical, it provided direction for future studies and emphasized the potential of combination therapies in antimicrobial treatment. A 2022 study demonstrated that combining phages with the probiotic *Lactobacillus* reuteri significantly alleviated colitis symptoms caused by *Salmonella* in a mouse model and promoted short-chain fatty acid (SCFA) production, suggesting the potential of combined strategies in modulating the microbiota (Wang et al., 2022). In 2024, a study found that in a mouse model of colitis, the use of fructooligosaccharides (FOS), Saccharomyces boulardii, and their combination yielded more significant anti-inflammatory effects and improved modulation of the gut microbiota (Wu et al., 2024a).

Additionally, host genetics also significantly influence the microbiome. For example, polymorphisms in the FMO3 gene affect the metabolism of TMAO, suggesting the potential for personalized microbial interventions based on genetic profiling (Moradzad et al., 2022).

#### 5.1.3 Metabolite-based intervention

Metabolites serve as key mediators in the crosstalk between the oral-gut axis and cardiovascular disease. Among them, trimethylamine N-oxide (TMAO), a metabolite produced by the gut microbiota through the metabolism of dietary choline and L-carnitine, has been shown to promote atherosclerosis (Zhu et al., 2020). Currently, researchers are developing inhibitors targeting key enzymes involved in TMAO production. For example, 3-nitrooxypropanol (3-NOP) inhibits *FMO3*, thereby blocking the conversion of TMA to TMAO. Early studies have demonstrated its significant TMAO-suppressing effects. Meanwhile, several small-molecule compounds have been developed to target choline-TMA lyase, reducing TMA production by up to 60% *in vitro* (Bollenbach et al., 2020). Another direction focuses on promoting the synthesis of beneficial metabolites, particularly SCFAs such as butyrate and propionate. Studies have shown that the intake of prebiotics such as inulin can enhance the abundance of *Bifidobacteria* and *Lactobacilli*, thereby boosting SCFA levels and reducing inflammation (van der Beek et al., 2018). In a clinical study, a 12-week high-inulin diet intervention significantly increased SCFA levels and improved systemic inflammation and blood pressure in patients with metabolic syndrome (Neyrinck et al., 2021).

Furthermore, postbiotics are being explored as a novel therapy, involving the direct use of microbial metabolic products such as purified butyrate or bacterial cell wall components to exert anti-inflammatory effects and protect vascular endothelium. For instance, a 2023 study found that butyrate, by activating *GPR43* receptors, reduced atherosclerotic plaque formation (Ma et al., 2023).

### 5.2 Non-invasive diagnostic biomarkers

New biomarkers are continuously emerging, including microbe-derived peptides and small RNAs. A 2022 study comparing the salivary microbiomes of patients with atherosclerotic cardiovascular disease (ACVD) and healthy individuals found significant differences in microbial composition (Kato-Kogoe et al., 2022). Another 2024 study analyzed the salivary microbiome and metabolome of carotid atherosclerosis patients in rural northeastern China and found that, compared to healthy controls, certain bacteria in the patients’ saliva were more abundant and associated with elevated levels of inflammatory markers (Wu et al., 2024b). The findings mentioned above highlight the potential diagnostic value of the salivary microbiome in cardiovascular diseases.

CVD is still the leading cause of morbidity and mortality worldwide. Framingham Risk Score (FRS) (Xu et al., 2025) and QResearch Risk Calculator (QRISK), as traditional CVD risk scores with certain limitations (Damen et al., 2016), easily neglect subclinical target organ damage and cannot accurately assess populations that can improve their health through lifestyle changes. Recent studies have explored new non-invasive risk markers involving serum biomarkers, cardiac imaging, and omics techniques to validate CVD risk stratification (Wu et al., 2024). Ina Nemet reported levels of gut microbiota-derived aromatic amino acid metabolites and their relationship with cardiovascular disease risk in two independent cohorts including 4000 samples from GeneBank in United States and 833 samples from LipidCardio in European in *European Heart Journal* (Nemet et al., 2023), showing that aromatic amino acid metabolites derived from gut microbiome can be used as alternative markers for risk assessment in patients with coronary artery disease (CAD) or suspected CAD (Stähli et al., 2023).

In terms of model construction, the integration of multi-omics data and artificial intelligence (AI) is advancing early prediction of cardiovascular diseases. Researchers have integrated gut microbiota, metabolites, and traditional clinical indicators to construct AI models predicting heart failure, achieving an area under the curve (AUC) exceeding 0.80 (Yang et al., 2025). SHapley Additive explanation (SHAP) value is used for analysis to interpret key features in oral-gut microbial prediction model (Vega García and Aznarte, 2020). Features with high positive SHAP values are helpful for disease prediction, while features with high negative SHAP values are helpful for health prediction. Gagandeep Marken developed a machine learning-based model for predicting CVD risk by integrating gut microbiome and clinical features, identifying key microbial taxa such as *Faecalibacterium prausnitzii* and *Bacteroides fragilis* as significant predictors of CVD (Marken and Naik, 2025).

**Figure 2:**
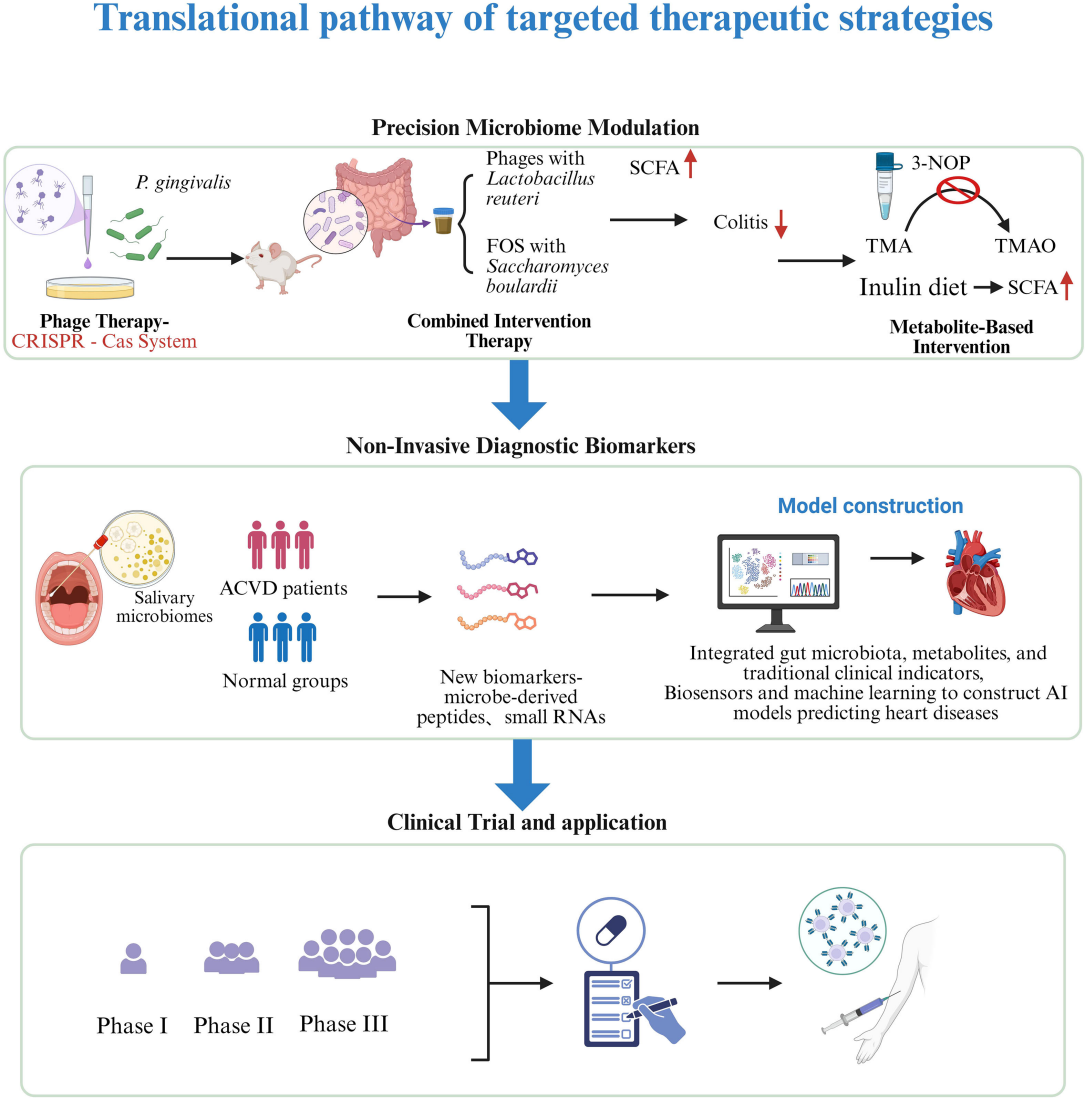
Translational pathway of targeted therapeutic strategies. From the discovery of phage therapy, metabolite intervention and Diagnostic biomarkers in basic research to preclinical research, clinical trials, regulatory approval, and ultimately clinical application to improve prognosis. Created in BioRender. Su, S. (2025) https://BioRender.com/1rcjibb.

Additionally, deep learning algorithms (e.g., neural networks) can analyze complex relationships among the microbiome, metabolites, and clinical variables. A 2024 study developed a system combining a nanobiosensor with a long short-term memory (LSTM) deep learning model to analyze circulating 16S rRNA microbial data in blood for CVD risk assessment. This model identified specific bacterial taxa associated with CVD, offering a novel approach for non-invasive early screening (Nazeer et al., 2024). Another 2025 study used the advanced LightGBM ensemble model to analyze gut microbiome data and accurately predict the presence of CVD (Marken and Naik, 2025). Moreover, models integrating biosensors and machine learning are also under development. For instance, a 2025 study found that combining non-invasive hyperspectral imaging of the oral microbiome with machine learning effectively predicted and diagnosed coronary artery disease (Li et al., 2025). These studies offer promising avenues for real-time screening of cardiovascular disease in clinical settings (**Figure 2**).

## 6 Challenges and future directions

### 6.1 Establishing causality and standardizing research methods

Although significant findings have emerged regarding the association between the oral-gut microbiota axis and cardiovascular diseases (CVDs), clarifying their causal mechanisms remains a major challenge. Currently, most studies are observational, drawing inferences based solely on associations between microbiota composition or microbial metabolites and cardiovascular risk factors. For example, oral pathogens including *Porphyromonas gingivalis* and *Fusobacterium nucleatum* have been consistently associated with the progression of atherosclerosis, but it remains unclear whether these microbial changes are causal contributors or merely consequences of disease pathology (Koren et al., 2011). Confounding factors like diet, smoking, and systemic inflammation further complicate interpretations.

To advance mechanistic understanding, well-designed randomized controlled trials (RCTs) should be prioritized. For instance, investigating the impact of targeted oral probiotics or phage therapies on cardiovascular outcomes in high-risk populations could provide more direct causal evidence. However, such trials face considerable challenges in recruiting suitable participants, defining clinical endpoints (e.g., changes in arterial plaque volume), and ensuring long-term compliance.

In addition, the standardization of technical methodologies is essential to improve research quality. Variations in sample types (saliva vs. subgingival plaque; feces vs. mucosal biopsies), sequencing techniques (16S rRNA vs. metagenomics), and data analysis pipelines have led to poor comparability across studies. There is an urgent need for standardized guidelines for microbiome research, akin to CONSORT or MIAME, to enhance reproducibility and cross-cohort integration.

Recently, humanized organoid systems have provided new models for validating causality. In 2025, researchers developed a vascular organoid co-culture system with microbes and found that *Klebsiella pneumoniae* could impair endothelial vasodilation function by disrupting the eNOS signaling pathway in endothelial cells, partially supporting the “marker hypothesis” that microbes may directly mediate vascular injury (Rehman et al., 2025).

### 6.2 Translational barriers and personalized medicine

Translating findings from oral-gut microbiome research into clinical interventions remains a major hurdle. Current therapies such as phage therapy and microbiota-targeted metabolic regulators are mostly in preclinical or early clinical stages and have not yet been widely integrated into CVD management. Personalized therapies, in particular, face challenges in scaling up production, controlling costs, and navigating regulatory approval.

To date, the U.S. Food and Drug Administration (FDA) has not approved any personalized phage preparations for routine human treatment. These are only accessible under specific circumstances via “compassionate use” or “single-use Investigational New Drug (IND)” pathways. For instance, the Center for Innovative Phage Applications and Therapeutics (IPATH) at the University of California, San Diego, provides personalized phage therapy under these pathways (https://time.com/5316516/phage-therapy-virus-san-diego/?utm_source=chatgpt.com), with patients typically covering the treatment costs themselves, which range from $10,000 to $75,000 (Is phage therapy FDA-approved for use in treating chronic infections).

Furthermore, the high complexity and interaction among host genetic background, microbial metabolic capabilities, and environmental factors limit the efficacy of one-size-fits-all approaches. For example, individual differences in *FMO3* gene expression influence the production of the gut-derived metabolite TMAO, thereby affecting CVD risk prediction and intervention outcomes, ultimately contributing to heterogeneity in treatment response (Robinson-Cohen et al., 2016; Wei et al., 2022). Liu et al. demonstrate that integrating polygenic risk scores (PTS) and gut microbiome can improve predictive value for heart disease in a longitudinal population-based cohort (C-statistics: 0.794, 95% CIs: 0.772–0.817) (Liu et al., 2024). Therefore, the future may lie in developing personalized medical strategies using multi-omics integration and artificial intelligence algorithms. For instance, by integrating metagenomic, metabolomic, and proteomic profiles to train predictive models, researchers could pre-identify individuals who are likely to benefit from specific microbiome interventions.

It is also worth noting that challenges remain regarding the delivery efficiency of microbiota-based therapeutics. Studies show that traditional orally administered probiotics have low survival rates before reaching the gut, significantly reducing their efficacy (Naissinger da Silva et al., 2021; Yang et al., 2023). Although gold nanomaterials have been widely used for targeted delivery, recent findings have confirmed their potential toxicity due to liver accumulation (Jakic et al., 2024; Zhang et al., 2024). Future research should shift toward safer, biodegradable alternatives. For example, mesoporous silica nanoparticles (MSNs) have been shown in a 2021 study to enhance probiotic stability and functionality under gastric acid conditions by serving as a protective layer (Centurion et al., 2021). Another 2024 study developed a hybrid microcapsule system based on natural biosilica and chitosan/shellac polymers, which demonstrated high probiotic survival rates in simulated gastrointestinal environments (Vona et al., 2024). These findings suggest that silica-based nanocarriers hold great promise in probiotic delivery systems, especially novel encapsulation technologies, although their *in vivo* biodegradability and long-term safety still require further investigation. Future studies should focus on the *in vivo* behavior of these materials to ensure their safety and efficacy in clinical applications.

### 6.3 Revealing complex mechanisms and crosstalk

Current understanding of the mechanisms by which the oral-gut axis contributes to cardiovascular disease remains preliminary. While key pathways such as inflammatory cascades and microbial metabolites (e.g., TMAO, short-chain fatty acids) have been identified, many questions remain. How do specific oral microbes cross systemic barriers to regulate gut immunity? How do gut microbial metabolites like secondary bile acids and indole derivatives affect endothelial function and myocardial metabolism? Functional mechanisms of these “non-mainstream” metabolites are still poorly understood.

Moreover, the bidirectional interactions between the oral and gut microbiota, along with their interplay with other systems such as the gut-brain axis and adipose tissue immune networks, add further complexity. Future studies should employ advanced technologies such as spatial transcriptomics and single-cell sequencing to accurately map the localization and functions of microbe-derived factors within the cardiovascular system. Longitudinal studies tracking microbiome changes over time and their relationship with disease progression will help elucidate dynamic mechanisms. Additionally, investigating the role of the oral-gut-cardiovascular axis in specific populations—such as the elderly, pregnant women, and individuals with comorbidities—may uncover unique mechanisms and therapeutic opportunities. For example, research has suggested that shifts in oral microbiota in elderly individuals may significantly alter gut inflammation, indicating that regulatory mechanisms of this axis may vary under different physiological conditions (Iwauchi et al., 2019).

### 6.4 Technical limitations and innovation

Existing technologies face several limitations in elucidating microbiome functions. 16S rRNA sequencing primarily enables microbial taxonomic identification but lacks the resolution to characterize functional activity; meanwhile, metabolomics often lacks the sensitivity to detect low-abundance or small signaling molecules. Cutting-edge tools such as next-generation multi-omics sequencing and single-cell multi-omics are helping to bridge these gaps. Microfluidic platforms like “oral-gut-on-a-chip” systems (Xiang et al., 2020; Makkar et al., 2023) can simulate local microbiota–immune interactions, supporting the forward-looking testing of therapeutic interventions.

Additionally, wearable and non-invasive monitoring devices are showing great promise in tracking the oral-gut axis in real time. For instance, smart toothbrushes equipped with sensors can analyze the composition of the oral microbiome (Oral microbiome research: Startup Spotlight: Innovations in Oral Health Using Microbiome Insights), while ingestible sensors have been developed to monitor gut metabolites (University of Maryland, 2025). These technologies enable continuous health monitoring and, when integrated with digital health platforms, can provide personalized feedback and intervention recommendations, helping individuals manage cardiovascular health by modulating their microbiota.

## 7 Conclusion

Research on the oral–gut microbiota axis has unveiled a previously underestimated yet critically important aspect of cardiovascular disease (CVD) pathogenesis. As highlighted in this comprehensive review, the intricate interplay between oral and gut microbiota—through microbial translocation, metabolic exchange, and immune signaling—substantially contributes to the pathogenesis and progression of atherosclerosis, hypertension, and heart failure.

Mounting evidence demonstrates that oral pathogens such as *Porphyromonas gingivalis* and *Fusobacterium nucleatum* can translocate and disrupt gut microbial homeostasis, triggering systemic inflammation and promoting atherogenesis (Koren et al., 2011; Hajishengallis, 2015). Microbial metabolites produced in the gut, particularly trimethylamine N-oxide (TMAO), play a pivotal role in cholesterol metabolism and plaque instability, further underscoring the impact of this axis on cardiovascular risk (Wang et al., 2011; Koeth et al., 2013; Tang and Hazen, 2014). In hypertension, the oral–gut axis has been implicated in mechanisms involving gut barrier dysfunction, endotoxemia, and renin–angiotensin system activation (Marques et al., 2017; Santisteban et al., 2017). Even in the complex pathology of heart failure, bidirectional interactions between microbial signals and cardiac function—mediated through inflammation or compensatory responses—underscore the axis’s broad pathophysiological relevance (Chen et al., 2023).

These mechanistic insights pave the way for novel therapeutic avenues. Precision microbiota modulation—through bacteriophage therapy, metabolite-targeting interventions, and the use of probiotics or prebiotics—offers a promising strategy for restoring microbial balance and attenuating disease progression (Wu et al., 2011). The emergence of non-invasive biomarkers, integrating oral microbiota profiles, serum metabolites, and clinical phenotypes, represents a significant advancement in early diagnosis and risk stratification of CVDs (Zhu et al., 2025). These developments not only expand the clinical toolbox for CVD management but also challenge conventional paradigms by emphasizing the integral role of microbiota in systemic health.

Nevertheless, translating current knowledge into widespread clinical practice remains fraught with challenges. Establishing causality through large-scale randomized controlled trials (RCTs), overcoming translational barriers to develop personalized interventions, and deciphering the complex molecular underpinnings of axis function remain formidable tasks. Moreover, technical limitations continue to hinder a full understanding of the microbiota’s dynamic and functional interactions, underscoring the need for continued methodological innovation.

Looking forward, research on the oral–gut microbiota axis in cardiovascular diseases holds immense promise. Future advances will rely on interdisciplinary collaborations among microbiologists, cardiologists, immunologists, and bioengineers to bridge existing knowledge gaps and transform prevention and treatment paradigms. With the aid of cutting-edge technologies and innovative methodologies, it may become possible to identify novel therapeutic targets and optimize personalized strategies—ultimately improving the quality of life for millions of patients worldwide. Once a relatively obscure field, the oral–gut microbiota axis is now emerging as a focal point of cardiovascular medicine, bringing hope for more effective management and prevention of CVDs.
